# Demystifying the spectral collapse in two-photon Rabi model

**DOI:** 10.1038/s41598-020-71637-z

**Published:** 2020-09-09

**Authors:** C. F. Lo

**Affiliations:** Department of Physics, Institute of Theoretical Physics, The Chinese University of Hong Kong, Shatin, New Territories Hong Kong

**Keywords:** Physics, Optical physics, Quantum physics

## Abstract

We have investigated the eigenenergy spectrum of the two-photon Rabi model at the critical coupling, particularly the special feature “spectral collapse”, by means of an elementary quantum mechanics approach. The eigenenergy spectrum is found to consist of both a set of discrete energy levels and a continuous energy spectrum. Each of these eigenenergies has a two-fold degeneracy corresponding to the spin degree of freedom. The discrete eigenenergy spectrum has a one-to-one mapping with that of a particle in a “Lorentzian function” potential well, and the continuous energy spectrum can be derived from the scattering problem associated with a potential barrier. The number of bound states available at the critical coupling is determined by the energy difference between the two atomic levels so that the extent of the “spectral collapse” can be monitored in a straightforward manner.

## Introduction

In 1963 Jaynes and Cummings^1^ introduced the quantum Rabi model as the simplest, yet non-trivial, model describing the interaction between radiation and matter by concentrating on the near-resonance linear coupling between a sinlge two-level atomic system and a quantized radiation mode $\left( \hbar =1\right)$:

1 $$\begin{aligned} H=\omega _{0}S_{z}+\omega a^{\dag }a+2\epsilon \left( a^{\dag }+a\right) S_{x}\ , \end{aligned}$$

where the radiation mode of frequency $\omega$ is described by the bosonic operators *a* and $a^{\dag }$, the two atomic levels separated by an energy difference $\omega _{0}$ are represented by the spin-half operators $S_{z}$ and $S_{x}$, and the atom-field coupling strength is measured by the positive parameter $\epsilon$. The various coupling regimes of the model can be specified in terms of the three model parameters. Due to recent technological advancement, the interest in this simple model has been increasing rapidly, and its applications are no longer limited to the weak coupling regime^2–14^. In addition, Braak’s discovery in 2011^15^ that the quantum Rabi model is exactly solvable further boosts the interest in the model.

Stimulated by the success of the quantum Rabi model, more and more people have paid special attention to extending and generalizing the model in order to explore new quantum effects. Among these generalizations, the quantum two-photon Rabi model is of particular interest $\left( \hbar =1\right)$:

2 $$\begin{aligned} H=\omega _{0}S_{z}+\omega a^{\dag }a+2\epsilon \left( a^{\dag 2}+a^{2}\right) S_{x}\ , \end{aligned}$$

and has been realized in many different experimental systems for a wide range of coupling strengths^16–26^. Unlike the one-photon counterpart, the two-photon generalization exhibits a particular feature, commonly known as the spectral collapse, which occurs when the coupling strength $\epsilon$ goes beyond a critical value $\epsilon _{c}\equiv \omega /2$. In 1998, via exact numerical diagonalization, Ng et al.^27^ first demonstrated that while the quantum two-photon Rabi model has a discrete eigenenergy spectrum for $\epsilon <\epsilon _{c}$, no normalizable eigenstate exists in the Hilbert space spanned by the photon number states for $\epsilon >\epsilon _{c}$. The authors pointed out that in the special case of $\omega _{0}=0$ the system corresponds to a quantum simple harmonic oscillator in the momentum space for $\epsilon <\epsilon _{c}$, whereas it represents an inverted harmonic potential barrier for $\epsilon >\epsilon _{c}$, and that this abrupt change in the fundemental nature of the system results in a transformation from a discrete eigenenergy spectrum to a continuous energy spectrum. In addition, at the critical coupling $\epsilon _{c}$ the system behaves like a free particle. For $\omega _{0}\ne 0$ the above analysis still holds for both $\epsilon <\epsilon _{c}$ and $\epsilon >\epsilon _{c}$ because the first term in Eq. (2) is a bounded operator. Nevertheless, the characteristic behaviour of the eigenstates at the critical coupling $\epsilon _{c}$ remains as a mystery.

Recently a number of theoretical studies on the spectral collapse^28–38^ of the model have indeed confirmed the results and observations of Ng et al.^27^. Nevertheless, our understanding of the quantum two-photon Rabi model at the critical coupling $\epsilon _{c}$ is still very limited because current theoretical approaches (both analytical and numerical) fail in dealing with the collapse point rigorously. While numerical methods (such as numerical exact diagonalization^27,35^ and the ones based upon spectral function and continued fraction^34^) suffer from the demand of a huge amount of computational power and unstable convergence, analytical analyses (like variational approximation^24^ and Braak’s G-function method^15,30–32^) are unable to approach the collapse point satisfactorily. For instance, as pointed out by Duan et al.^33^, the G-function method seems to suggest that the eigenenergy spectrum at the critical coupling $\epsilon _{c}$ consists of a discrete part in addition to a continuum: the ground state is always separated from the continuum by a finite excitation gap, ruling out a quantum phase transition in the usual sense, whereas the perturbation theory predicts the vanishing of the gap to all orders, demonstrating its non-perturbative nature. In addition, performing a numerical study of both the spectral functions and survival probabilities based upon a continued fraction approach, Lupo et al.^34^ identifies a signal suggesting that there is a remaining relevant discrete point in the spectrum.

Accordingly, the crucial contribution of our work is to solve this mystery completely. It is found that at the critical coupling $\epsilon _{c}$ the eigenenergy spectrum of the two-photon Rabi model consists of both a set of discrete energy levels and a continuous energy spectrum. The discrete eigenenergy spectrum has a one-to-one mapping with that of a particle in a “Lorentzian function” potential well whose eigenspectrum can be easily determined by an elementary quantum mechanics approach and the continuous energy spectrum can be derived from the scattering problem associated with a potential barrier. Without loss of generality, we set the energy unit such that $\omega =1$ for simplicity in the following analysis.

## Two-photon Rabi model

As shown in Ng et al.^27^, we apply the unitary transformation

3 $$\begin{aligned} R=\exp \left\{ -\frac{i\pi }{2}\left( S_{x}-\frac{1}{2}\right) a^{\dag }a\right\} \end{aligned}$$

to transform the Hamiltonian *H* in Eq. (2) to

4 $$\begin{aligned} {\tilde{H}}=R^{\dag }HR=\omega _{0}\cos \left( \frac{\pi }{2}a^{\dag }a\right) S_{z}+\omega _{0}\sin \left( \frac{\pi }{2}a^{\dag }a\right) S_{y}+a^{\dag }a+\epsilon \left( a^{\dag 2}+a^{2}\right) \ . \end{aligned}$$

Defining the “position” and “momentum” operators of the boson mode as

5 $$\begin{aligned} x=\frac{1}{\sqrt{2}}\left( a+a^{\dag }\right) \end{aligned}$$

and

6 $$\begin{aligned} p=\frac{1}{i\sqrt{2}}\left( a-a^{\dag }\right) \end{aligned}$$

respectively, the transformed Hamiltonian ${\tilde{H}}$ is given by

7 $$\begin{aligned} {\tilde{H}}=\omega _{0}\cos \left( \frac{\pi }{2}\left[ H_{0}-\frac{1}{2} \right] \right) S_{z}+\omega _{0}\sin \left( \frac{\pi }{2}\left[ H_{0}- \frac{1}{2}\right] \right) S_{y}+\left( H_{0}-\frac{1}{2}\right) -\epsilon \left( p^{2}-x^{2}\right) \ , \end{aligned}$$

where

8 $$\begin{aligned} H_{0}=\frac{p^{2}}{2}+\frac{x^{2}}{2}=a^{\dag }a+\frac{1}{2} \end{aligned}$$

is the Hamiltonian of a quantum simple harmonic oscillator of unit mass. At the critical coupling $\epsilon _{c}\equiv 1/2$, Eq. (7) is reduced to

9 $$\begin{aligned} {\tilde{H}}=\omega _{0}\cos \left( \frac{\pi }{2}\left[ H_{0}-\frac{1}{2} \right] \right) S_{z}+\omega _{0}\sin \left( \frac{\pi }{2}\left[ H_{0}- \frac{1}{2}\right] \right) S_{y}+x^{2}-\frac{1}{2}\ . \end{aligned}$$

It is not difficult to see that within the subspace of even number states of $H_{0}$ the transformed Hamiltonian ${\tilde{H}}$ becomes

10 $$\begin{aligned} {\tilde{H}}_{e}=\omega _{0}\cos \left( \frac{\pi }{2}\left[ H_{0}-\frac{1}{2} \right] \right) S_{z}+x^{2}-\frac{1}{2}\ , \end{aligned}$$

whereas within the subspace of odd number states we have

11 $$\begin{aligned} {\tilde{H}}_{o}=\omega _{0}\sin \left( \frac{\pi }{2}\left[ H_{0}-\frac{1}{2} \right] \right) S_{y}+x^{2}-\frac{1}{2}\ . \end{aligned}$$

Obviously, in both cases the spin degree of freedom and the boson mode are decoupled.

The eigenstates of ${\tilde{H}}_{e}$ are simply given by the states $\left\{ |M_{z}\rangle |\phi _{e}\rangle \right\}$, where $|M_{z}\rangle$ is an eigenstate of the spin operator $S_{z}$ and $|\phi _{e}\rangle$ is an eigenstate of even parity of the one-body Hamiltonian $h_{e}$:

12 $$\begin{aligned} h_{e}=M_{z}\omega _{0}\sqrt{i}\exp \left( -\frac{i\pi }{2}H_{0}\right) +x^{2}-\frac{1}{2} \end{aligned}$$

for $M_{z}=\pm 1/2$. In the coordinate space the eigenvalue equation of $h_{e}$ reads

13 $$\begin{aligned} E\phi _{e}\left( x\right)=\,& {} \left\{ M_{z}\omega _{0}\sqrt{i}\exp \left( - \frac{i\pi }{2}H_{0}\right) +x^{2}-\frac{1}{2}\right\} \phi _{e}\left( x\right) \nonumber \\=\,& {} \left( x^{2}-\frac{1}{2}\right) \phi _{e}\left( x\right) +M_{z}\omega _{0} \sqrt{i}\int _{-\infty }^{\infty }\frac{dy}{\sqrt{i2\pi }}\exp \left\{ -ixy\right\} \phi _{e}\left( y\right) \nonumber \\=\,& {} \left( x^{2}-\frac{1}{2}\right) \phi _{e}\left( x\right) +M_{z}\omega _{0} {\tilde{\phi }}_{e}\left( x\right) \ , \end{aligned}$$

where *E* denotes the eigenenergy and

14 $$\begin{aligned} {\tilde{\phi }}_{e}\left( p\right) =\int _{-\infty }^{\infty }\frac{dx}{\sqrt{ 2\pi }}\exp \left\{ -ipx\right\} \phi _{e}\left( x\right) \end{aligned}$$

is the Fourier transform of $\phi _{e}\left( x\right)$. Here we have made use of the fact that

15 $$\begin{aligned} \exp \left( -itH_{0}\right) \phi _{e}\left( x\right) =\int _{-\infty }^{\infty }dyK\left( x,t;y\right) \phi _{e}\left( y\right) \end{aligned}$$

where $K\left( x,t;y\right)$ is the propagator of $H_{0}$ defined by

16 $$\begin{aligned} K\left( x,t;y\right) =\frac{1}{\sqrt{i2\pi \sin \left( t\right) }}\exp \left\{ -\frac{\left( x^{2}+y^{2}\right) \cos \left( t\right) -2xy}{i2\sin \left( t\right) }\right\} \ . \end{aligned}$$

Similarly, in the momentum space the eigenvalue equation of $h_{e}$ is given by

17 $$\begin{aligned} E{\tilde{\phi }}_{e}\left( p\right) =\left( -\frac{d^{2}}{dp^{2}}-\frac{1}{2} \right) {\tilde{\phi }}_{e}\left( p\right) +M_{z}\omega _{0}\phi _{e}\left( p\right) \ . \end{aligned}$$

On the other hand, the eigenstates of ${\tilde{H}}_{o}$ consist of the states $\left\{ M_{y}\rangle |\phi _{o}\rangle \right\}$, where $|M_{y}\rangle$ is an eigenstate of the spin operator $S_{y}$ and $|\phi _{o}\rangle$ is an eigenstate of odd parity of the one-body Hamiltonian $h_{o}$:

18 $$\begin{aligned} h_{o}=M_{y}\omega _{0}i\sqrt{i}\exp \left( -\frac{i\pi }{2}H_{0}\right) +x^{2}-\frac{1}{2} \end{aligned}$$

for $M_{y}=\pm 1/2$. The eigenvalue equations of $h_{o}$ in both coordinate space and momentum space are given by

19 $$\begin{aligned} E\phi _{o}\left( x\right) =\left( x^{2}-\frac{1}{2}\right) \phi _{o}\left( x\right) +iM_{y}\omega _{0}{\tilde{\phi }}_{o}\left( x\right) \end{aligned}$$

and

20 $$\begin{aligned} E{\tilde{\phi }}_{o}\left( p\right) =\left( -\frac{d^{2}}{dp^{2}}-\frac{1}{2} \right) {\tilde{\phi }}_{o}\left( p\right) -iM_{y}\omega _{0}\phi _{o}\left( p\right) \ , \end{aligned}$$

respectively.

Eliminating $\phi _{e}\left( x\right)$ from Eqs. (13) and (17) as well as $\phi _{o}\left( x\right)$ from Eqs. (19) and (20) yields

21 $$\begin{aligned} \left( E+\frac{1}{2}\right) \left\{ \begin{array}{c} {\tilde{\phi }}_{e}\left( p\right) \\ {\tilde{\phi }}_{o}\left( p\right) \end{array} \right\} =-\frac{d^{2}}{dp^{2}}\left\{ \begin{array}{c} {\tilde{\phi }}_{e}\left( p\right) \\ {\tilde{\phi }}_{o}\left( p\right) \end{array} \right\} +\frac{\omega _{0}^{2}}{E+\frac{1}{2}-p^{2}}\left\{ \begin{array}{c} M_{z}^{2}{\tilde{\phi }}_{e}\left( p\right) \\ M_{y}^{2}{\tilde{\phi }}_{o}\left( p\right) \end{array} \right\} \ . \end{aligned}$$

Since $M_{z}^{2}=M_{y}^{2}=1/4$, it is clear that ${\tilde{\phi }}_{e}\left( p\right)$ and ${\tilde{\phi }}_{o}\left( p\right)$ simply denote the even-parity and odd-parity solutions of the eigenvalue equation

22 $$\begin{aligned} \left( E+\frac{1}{2}\right) {\tilde{\phi }}\left( p\right) =-\frac{d^{2}\tilde{ \phi }\left( p\right) }{dp^{2}}+\frac{\left( \omega _{0}/2\right) ^{2}}{E+ \frac{1}{2}-p^{2}}{\tilde{\phi }}\left( p\right) \ , \end{aligned}$$

respectively. For $$E+1/2<0$$, we introduce the parameter $$\kappa =$$$$\sqrt{ \left| E+1/2\right| }$$ and define a new variable *q* $$=p/\kappa$$ such that Eq. (22) can be expressed as

23 $$\begin{aligned} -\kappa ^{4}{\tilde{\phi }}\left( q\right) =-\frac{d^{2}{\tilde{\phi }}\left( q\right) }{dq^{2}}-\frac{\left( \omega _{0}/2\right) ^{2}}{1+q^{2}}\tilde{ \phi }\left( q\right) \ , \end{aligned}$$

which is the time-independent Schrödinger equation of the bound state problem associated with a “Lorentzian function” potential well. For $$E+1/2>0$$, in terms of the parameter $$k=$$$$\sqrt{E+1/2}$$ and the new variable $${\bar{q}}$$$$=p/k$$, Eq. (22) becomes

24 $$\begin{aligned} k^{4}{\tilde{\phi }}\left( {\bar{q}}\right) =-\frac{d^{2}{\tilde{\phi }}\left( \bar{q }\right) }{d{\bar{q}}^{2}}+\frac{\left( \omega _{0}/2\right) ^{2}}{1-{\bar{q}} ^{2}}{\tilde{\phi }}\left( {\bar{q}}\right) \ , \end{aligned}$$

which is the time-independent Schrödinger equation of the scattering state problem associated with the potential barrier: $\left( 1-{\bar{q}} ^{2}\right) ^{-1}$ that is singular at ${\bar{q}}=\pm 1$.

Accordingly, at the critical coupling $\epsilon _{c}$ the system not only has a set of discrete eigenenergies but it also has a continuous energy spectrum. In Eq. (23) the parameter $\omega _{0}$ specifies the depth of the “Lorentzian function” potential well and determines the number of bound states available. It is well known that there is at least one bound state for $\omega _{0}>0$. On the other hand, in Eq. (24) the parameter $\omega _{0}$ specifies the magnitude of the potential barrier. Moreover, the disappearance of spin eigenvalues in Eq. (22) implies that each eigenstate is doubly degenerate.

## Conclusion

In this communication we have shown that at the critical coupling $\epsilon _{c}$ the eigenenergy spectrum of the two-photon Rabi model consists of both a set of discrete energy levels and a continuous energy spectrum, and that each of these eigenenergies has a two-fold degeneracy corresponding to the spin degree of freedom. The discrete eigenenergy spectrum has a one-to-one mapping with that of a particle in a “Lorentzian function” potential well, and the continuous energy spectrum can be derived from the scattering problem associated with a potential barrier. It is obvious that whilst setting $\omega _{0}=0$ in Eq. (24) results in the time-independent Schrödinger equation of a free particle, Eq. (23) is reduced to one with no admissible solution. Since both Eqs. (23) and (24) cannot be solved in closed form, we need to resort to numerical methods. As a result, it can be concluded that the two-photon Rabi model has three different regimes: $\left( 1\right)$ a purely discrete eigenenergy spectrum for $\epsilon <\epsilon _{c}$, $\left( 2\right)$ a purely continuous energy spectrum for $\epsilon >\epsilon _{c}$, and $\left( 3\right)$ a combination of a set of discrete energy levels and a continuous energy spectrum at $\epsilon =\epsilon _{c}$. The number of bound states available at the critical coupling $\epsilon _{c}$ can be controlled by adjusting the parameter $\omega _{0}$, implying that the extent of the spectral collapse can be monitored in a straightforward manner.

Furthermore, Ng et al.^39,40^ has demonstrated that spectral collapse also appears in two other generalizations of the quantum Rabi model, namely the intensity-dependent Rabi model $\left( \hbar =1\right)$:

25 $$\begin{aligned} H=\omega _{0}S_{z}+\omega a^{\dag }a+2\epsilon \left( \sqrt{a^{\dag }a} a^{\dag }+a\sqrt{a^{\dag }a}\right) S_{x}\ , \end{aligned}$$

and the two-mode two-photon Rabi model $\left( \hbar =1\right)$:

26 $$\begin{aligned} H=\omega _{0}S_{z}+\omega \left( a_{1}^{\dag }a_{1}+a_{2}^{\dag }a_{2}\right) +2\epsilon \left( a_{1}^{\dag }a_{2}^{\dag }+a_{1}a_{2}\right) S_{x}\ . \end{aligned}$$

Analogous to the two-photon Rabi model, the intensity-dependent Rabi model exhibits spectral collapse for the coupling strength $\epsilon$ being larger than a critical value $\epsilon _{c}\equiv \omega /2$, whilst in the two-mode two-photon Rabi model spectral collapse occurs at the critical coupling $\epsilon _{c}\equiv \omega$. This similiarity arises from the fact that the three generalizations of the quantum Rabi model share the same $SU\left( 1,1\right)$ dynamical symmetry^41^. Hence, we believe that a similar approach can be applied to tackle the spectral collapse problem of these two models.
